# Effect of high‐flow nasal oxygen on postoperative oxygenation in obese patients: A randomized controlled trial

**DOI:** 10.1002/hsr2.616

**Published:** 2022-04-18

**Authors:** Jacob Rosén, Peter Frykholm, Diddi Fors

**Affiliations:** ^1^ Department of Surgical Sciences, Section of Anaesthesiology and Intensive Care Medicine Uppsala University Uppsala Sweden

**Keywords:** bariatric surgery, high‐flow nasal oxygen, obese, oxygenation, postoperative hypoxemia

## Abstract

**Background and Aim:**

Postoperative hypoxemia is common after general anesthesia in obese patients. We investigated if early application of high‐flow nasal oxygen (HFNO) improved postoperative oxygenation in obese patients compared with standard oxygen therapy following general anesthesia for laparoscopic bariatric surgery.

**Methods:**

This was an open labeled randomized controlled trial conducted at a university hospital in Sweden between October 23, 2018 and February 11, 2020. The study was performed as a substudy within a previously published trial. After ethics committee approval and written informed consent, 40 obese patients (body mass index [BMI] ≥ 35 kg m^−2^) scheduled for laparoscopic bariatric surgery were randomized to receive oxygen using a standard low‐flow nasal cannula (NC group) or HFNO at 40 L min^−1^ (HF group) immediately upon arrival to the post‐anesthesia care unit. Flow rate (NC group) or FiO_2_ (HF group) was titrated to reach an initial SpO_2_ of 95%–98% after which settings were left unchanged. The primary outcome was PaO_2_ at 60 min following postoperative baseline values. Secondary outcomes included PaCO_2_, SpO_2_, hemodynamic variables, and patient self‐assessed discomfort.

**Results:**

Thirty‐four patients were available for analysis. PaO_2_ was similar between groups at postoperative baseline. After 60 min, PaO_2_ had increased to 12.6 ± 2.8 kPa in the NC group (*n* = 15) and 14.0 ± 2.7 kPa in the HF group (*n* = 19); (mean difference 1.4 kPa, 95% confidence interval −0.6 to 3.3; *p* = 0.16). There were no differences in PaCO_2_, hemodynamic variables, or self‐assessed discomfort between groups after 60 min.

**Conclusion:**

In obese patients, HFNO did not improve postoperative short‐term oxygenation compared with standard low‐flow oxygen following general anesthesia for laparoscopic bariatric surgery.

## 1 INTRODUCTION

After general anesthesia, disconnection from the ventilator circuit results in loss of positive airway pressure that may lead to lung derecruitment and atelectasis‐related shunt,^1^, ^2^ especially in obese patients.^3^, ^4^ This is a major cause of postoperative hypoxemia and the cause of considerable postoperative morbidity^5^, ^6^; hence there is a need for preventive strategies.^7^

In patients with hypoxemic respiratory failure after abdominal surgery, the application of continuous positive airway pressure (CPAP) reduces the need for intubation and mechanical ventilation.^8^ Although high‐flow nasal oxygen (HFNO) likewise increases airway pressure and therefore may prevent atelectasis formation, early application of this technique in patients with body mass index (BMI) < 35 kg m^−2^ failed to reduce the incidence of postoperative hypoxemia after major abdominal surgery compared with standard low‐flow oxygen.^9^ Furthermore, after cardiothoracic surgery, HFNO did not improve atelectasis formation on chest X‐ray compared with standard nasal cannula.^10^

In obese patients undergoing laparoscopic bariatric surgery, CPAP improves postoperative oxygenation, and immediate application after extubation maintains respiratory function better than application in the post‐anesthesia care unit (PACU).^11^, ^12^ Contrary to the findings in studies of patients with normal BMI, HFNO has been reported to reduce the risk of postoperative hypoxemia in obese patients compared with standard oxygen supplementation when HFNO was applied in the operating room before extubation and maintained during transport to the PACU.^13^ Although this strategy eliminates time without positive airway pressure and presumably reduces the risk of lung derecruitment, the transport of patients with HFNO remains challenging in clinical practice. The effect of HFNO on postoperative oxygenation in obese patients when applied early instead of immediately after extubation has not been well characterized.

We hypothesized that HFNO would improve postoperative oxygenation in obese patients compared with standard low flow oxygen therapy when applied at arrival to the PACU. The primary aim of this trial was to compare oxygenation using these two strategies during the first postoperative hour in obese patients recovering from general anesthesia for laparoscopic bariatric surgery.

## 2 METHODS

### 2.1 Study design, setting, and ethical statement

This was a randomized controlled trial conducted at a university hospital in Sweden between October 23, 2018 and February 11, 2020. The study complied with the Declaration of Helsinki and Good Clinical Practice. Ethical permission was granted by the Regional Ethical Committee of Uppsala, Sweden, on April 4, 2018. Written informed consent was obtained before induction of anesthesia in all patients. The protocol was registered a priori at the ISRCTN registry (#ISRCTN37375068) October 19, 2018 (isrctn.com) and the study adhered to the CONSORT guidelines for reporting randomized trials.

### 2.2 Participants

Adult patients, aged 18–60 years, scheduled for elective laparoscopic bariatric surgery were eligible for study inclusion if they had a BMI ≥ 35 kg m^−2^. Patients were excluded if they had an ASA Class > 2 (not assessed by BMI), chronic obstructive pulmonary disease or asthma causing limitations in everyday activities, heart failure with clinical symptoms equivalent to New York Heart Association functional classification > 2, restrictive lung disease with > 20% reduction in total lung capacity, allergy to anesthetic agents used in the study, or if they could not comprehend oral or written information.

### 2.3 Study prerequisites, randomization, and masking

This trial was performed as a sub‐study within a previously published trial^14^ in which patients were randomized to receive preoxygenation with a FiO_2_ of 1.0 using face mask and positive end‐expiratory pressure (PEEP) of 7 cmH_2_O or HFNO with 70 L min^−1^ before induction of anesthesia. Patients randomized to face‐mask with PEEP were also allocated to postoperative oxygen supplementation with a standard nasal cannula (NC group), whereas patients randomized to preoxygenation with HFNO were allocated to receive postoperative oxygen supplementation with HFNO (HF group). Randomization was performed before induction of anesthesia in an allocation ratio of 1:1 and a block size of two by opening sealed opaque sequentially numbered envelopes. There was no blinding.

### 2.4 Interventions

Patients in the NC group received oxygen at 2 L min^−1^ using a standard nasal cannula approximately corresponding to a FiO_2_ of 0.30,^15^ whereas patients in the HF group received HFNO (POINT® high flow system, Armstrong Medical) applied immediately upon the arrival to the PACU with a flow rate of 40 L min^−1^ and a FiO_2_ of 0.30. Flow rate (NC group) or FiO_2_ (HF group) was titrated to reach a SpO_2_ of 95%–98% and settings were thereafter left unchanged during the study period. The PACU was located near the operation room.

### 2.5 Anesthesia management

All patients were premedicated with Paracetamol 1 g and Oxycodone 5 mg orally approximately 1 h before anesthesia. A radial arterial line was inserted with ultrasound guidance. Anesthesia was induced with 2 µg kg^−1^ Fentanyl based on predicted body weight, a target‐controlled infusion of Propofol, and 0.6 mg kg^−1^ Rocuronium bromide based on lean body weight. After intubation, anesthesia was maintained with a target‐controlled infusion of Propofol and Remifentanil. Patients were mechanically ventilated using pressure‐regulated volume control mode with a recommended PEEP of 7 cmH_2_0 and tidal volumes of 6–8 ml kg^−1^. Definitive ventilator settings and dosing of anesthetic agents were left at the discretion of the attending anesthetist.

At the end of the surgery, a standardized lung recruitment maneuver was performed per routine in all patients to reduce postoperative pain by augmenting exsufflation of residual pneumoperitoneum^16^ and to reduce atelectasis.^17^, ^18^ The ventilator mode was changed to pressure control with inspiration:expiration ratio 1:1, respiratory frequency 6 min^−1^, PEEP 15–17 H_2_O, and inspiratory pressure 15 cmH_2_O for 1 min before the abdominal surgical ports were removed.

Neuromuscular block was reversed targeting a TOF ≥ 90% using either Neostigmine and Atropine or Sugammadex as appropriate. Patients were extubated in the operating room to spontaneous breathing and positioned in a semi‐recumbent position. Supplemental oxygen was delivered through a standard nasal cannula at a flow rate of 2–4 L min^−1^ after extubation and maintained during transport to the PACU. Immediately on arrival in the PACU, HFNO was started in the HF group, while the standard nasal cannula was left in place in the NC group.

### 2.6 Outcomes

The primary outcome was PaO_2_ 60 min following completed titration of allocated oxygen supplementation in the PACU. Secondary outcomes were SpO_2_, PaCO_2_, pH, noninvasive blood pressure (NIBP), heart rate at 60 min. Further secondary outcomes were incidence of SpO_2_ < 90% and the level of comfort using either intervention.

### 2.7 Measurements

Arterial blood was sampled 10 min after completed titration of allocated oxygen therapy in the PACU, and at 30 and 60 min thereafter. Blood samples were analyzed with a point of care device (Abbot i‐STAT® 1, Abbott Laboratories). SpO_2_, NIBP, and heart rate were recorded with a standard bedside monitor (Dräger Infinity Delta® monitor, Dräger Medical System Inc.) at the same time points as arterial blood sampling. Duration and incidence of SpO_2_ < 90% and information on opioid requirements were extracted from the electronic medical record (Metavision®). The level of discomfort in regard to the interventions was assessed using a four‐step ordinal scale defined as none, mild, moderate, or severe.

### 2.8 Sample size calculation

The sample size calculation was performed for the original trial.^19^ The sample size for the present study may therefore be considered a convenience sample. However, to confirm adequate power in the current investigation, a post hoc estimation of the minimal detectable effect was performed. Considering the sample size and the dispersion estimates of the final analysis, the present study had an 80% power to detect a 20% difference in PaO_2_ at 60 min after intervention initiation at *α* of 0.05.

### 2.9 Statistical analysis

Analyses were performed with Microsoft Excel and R (version 4.0.2) with the R package *Rcmdr* (R Commander. R package version 2.7‐0). Normality of data was assessed with quintile‐quintile plots and confirmed with Shapiro–Wilk test. Continuous data were presented as mean ± standard deviations (*SD*) or median (interquartile range [IQR]) with or without range as appropriate. Categorical data were presented as numbers and percentages. The primary outcome was analyzed with unpaired *t* test. Secondary outcomes were analyzed with unpaired *t* test or Mann–Whitney's test as appropriate. Fisher exact test was used to compare categorical variables. The effect size for mean differences was estimated with 95% confidence intervals (95% CI). Secondary analyses were not corrected for multiple testing and imputation was not performed for missing data. All tests were two‐sided and *p* values <0.05 were considered statistically significant.

## 3 RESULTS

**Figure 1:**
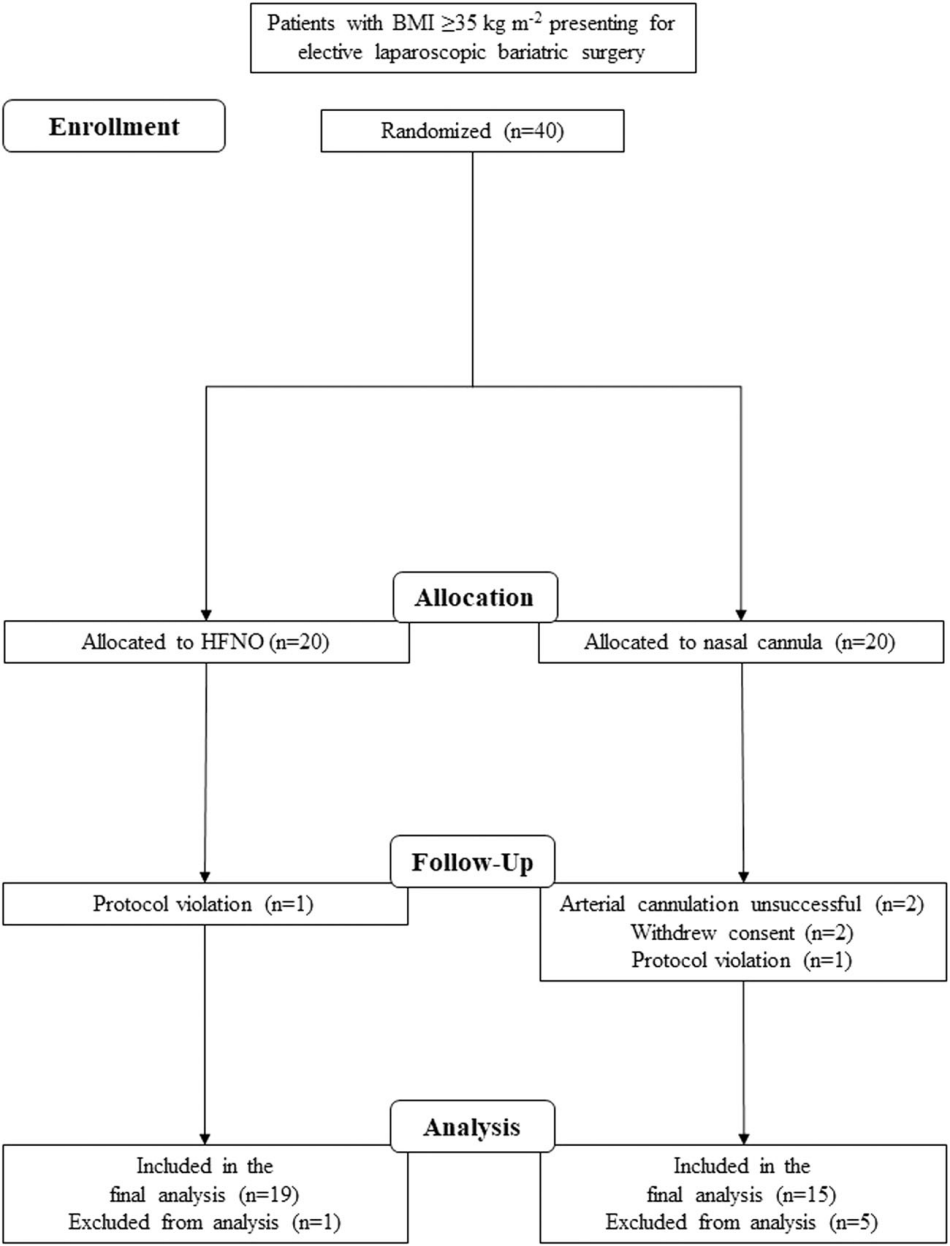
Consolidated Standards of Reporting Trials (CONSORT) flow chart of enrolled, randomized, and analyzed participants. BMI, body mass index; HFNO, high‐flow nasal oxygen

Of 40 randomized patients, 15 patients were available for final analysis in the NC group and 19 patients in the HF group (Figure 1). In the NC group, two subjects withdrew consent and arterial access could not be achieved in two additional subjects. One additional patient in each group was excluded due to a protocol violation. Patients in the HF group were a mean of 5 years older compared with controls, and five (26%) patients in the HF group were active or previous smokers compared with 2 (13%) in the NC group. There were furthermore patients with asthma in the HF group, however, all had mild disease and were asymptomatic on the day of the study. Subject characteristics and intraoperative respiratory measurements were otherwise similar between groups (Table 1).

**Table 1:** Subject characteristics, baseline measurements before general anesthesia, details on intraoperative characteristics and respiratory measurements, and settings of postoperative oxygen supplementation

	NC group (*n* = 15)	HF group (*n* = 19)
Age (years)	39 ± 10	44 ± 11
Sex (female)	14 (93)	16 (84)
Height (cm)	167 ± 6	166 ± 9
Weight (kg)	112 ± 9	110 ± 9
BMI (kg m^−2^)	40.0 ± 2.8	40.2 ± 4.2
BMI 35–40 *n* (%)	7 (47)	9 (47)
BMI > 40 *n* (%)	8 (53)	10 (53)
ASA 1/2	6/9	3/16
OSAS	2 (13)	3 (16)
Smoker	2 (13)	5 (26)
Ongoing	0 (0)	1 (5)
Previous	2 (13)	4 (21)
Asthma	2 (13)	6 (32)
Hypertension	3 (20)	4 (21)
Diabetes	1 (7)	1 (5)
Measurements on room air before anesthesia		
PaO_2_ (kPa)	11.7 ± 1.4	11.7 ± 1.8
SpO_2_ (%) median [range]	99 [98–100]	100 [98–100]
PaCO_2_ (kPa)	4.82 ± 0.25	4.92 ± 0.42
Intraoperative characteristics and respiratory measurements		
Duration of surgery (min)	57 ± 12	53 ± 10
PEEP (cmH_2_O) median (IQR) [range]	7 (7–8) [5–10]	7 (7–8) [5–10]
Dynamic compliance before standardized intraoperative recruitment maneuverer (ml/cmH_2_O)	38 ± 8	35 ± 10
Dynamic compliance after standardized intraoperative recruitment maneuverer (ml/cmH_2_O)	54 ± 17	60 ± 12
Initial postoperative settings		
Flow (L min^−1^), median (IQR) [range]	2 (2–2) [1–3]	40 (40–40) [20–40]
FiO_2_, median (IQR) [range]	n.a.	0.30 (0.30–0.30) [0.30–0.40]

*Note*: Data are presented as mean ± standard deviation for continuous variables and *n* (%) for categorical variables if not stated otherwise.

Abbreviations: BMI, body mass index; HF, high flow; IQR, interquartile range; NC, nasal cannula; OSAS, obstructive sleep apnea syndrome; PEEP, positive end‐expiratory pressure.

### 3.1 Primary outcome

**Figure 2:**
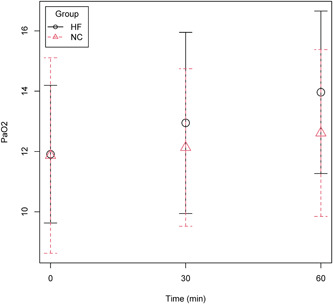
Graph presenting mean and standard deviations of postoperative PaO_2_ at baseline, 30 and 60 min in patients randomized to standard nasal cannula (NC group), *n* = 15, or high‐flow nasal oxygen (HF group), *n* = 19, for oxygen supplementation after laparoscopic bariatric surgery. PaO_2_ was similar at baseline and increased to 12.6 ± 2.8 kPa in the NC group and 14.0 ± 2.7 kPa in the HF group at 60 min (mean difference 1.4 kPa, 95% CI −0.6 to 3.3; *p* = 0.16).

At baseline, 10 min after completed titration of the assigned postoperative oxygen therapy PaO_2_ was 11.9 ± 3.2 kPa in the NC group and 11.9 ± 2.3 kPa in the HF group. After 60 min, PaO_2_ had increased to 12.6 ± 2.8 kPa in the NC group and 14.0 ± 2.7 kPa in the HF group (Figure 2), which was not statistically significant (mean difference 1.4 kPa, 95% CI −0.6 to 3.3; *p* = 0.16). Relative change from baseline was 8 ± 15% in the NC group and 19 ± 21% in the HF group (mean difference 11%, 95% CI −1.9 to 23; *p* = 0.09). Three (20%) patients in the NC group and three (16%) patients in the HF group decreased in PaO_2_ at 60 min compared with baseline (*p* = 0.75).

### 3.2 Secondary outcomes

There were no differences in SpO_2_, PaCO_2_ or hemodynamic variables between groups (Table 2). In the NC group, 14 (88%) and 2 (12%) patients reported no or mild discomfort respectively, and in the HF group 18 (90%), 1 (5%), and 1 (5%) patients reported no, mild, and moderate discomfort respectively (*p* = 0.77). Patients received a median (IQR) of 5 (3–6) mg intravenous oxycodone in the NC group and 6 (3–8) mg in the HF group (*p* = 0.60).

**Table 2:** Secondary outcomes

	Baseline postoperative measurements		60 min postoperative measurements		Mean difference (95% CI)	*p* Value
	NC	HF	NC	HF		
SpO_2_ (%)	97 [96–99]	96 [96–98]	98 [96–99]	99 [98–100]		0.10
PaCO_2_ (kPa)	6.3 ± 0.8	6.4 ± 1.0	5.7 ± 0.7	5.8 ± 0.8	0.1 (−0.40 to 0.66)	0.62
HR (min^−1^)	82 ± 9	80 ± 12	68 ± 12	71 ± 14	3 (−6 to 12)	0.49
SBP (mmHg)	134 ± 24	130 ± 17	138 ± 22	131 ± 18	−7 (−20 to 8)	0.37
DBP (mmHg)	80 ± 9	84 ± 14	82 ± 10	76 ± 15	−6 (−15 to 2)	0.14

*Note*: Data are presented as mean ± standard deviation or median [interquartile range] as appropriate. NC, Patients allocated to standard nasal cannula (*n* = 16). HF, patients allocated to high‐flow nasal oxygen (*n* = 20).

Abbreviations: DBP, diastolic blood pressure; HR, heart rate; RR, respiratory rate; SBP, systolic blood pressure.

### 3.3 Adverse events

Five (15%) patients had SpO_2_ lower than 90% during the study period. Two patients in the NC and HF groups respectively had one shorter (less than 2 min) episode of desaturation below 90%. None of these patients had a SpO_2_ of less than 85% at any timepoint. One patient in the HF group had 10 desaturation episodes below 90% and four of these were less than 85%.

## 4 DISCUSSION

The main finding of this study was that early application of postoperative HFNO after general anesthesia for laparoscopic bariatric surgery failed to improve oxygenation compared with standard nasal cannula in patients with BMI ≥ 35 kg m^−2^.

HFNO generates positive airway pressure directly correlated to flow rate and inversely related to mouth opening.^20^, ^21^, ^22^ Furthermore, HFNO improves respiratory mechanics and gas exchange due to expansion of the functional residual capacity.^23^, ^24^ These physiological findings suggest that HFNO could be beneficial for postoperative oxygen supplementation in obese patients. However, the airway pressures generated by HFNO are moderate at best^22^ and if patients are breathing through the mouth, the PEEP effect is nearly nullified.^20^ This may in part explain the neutral findings of our study.

Obese patients have reduced FRC at baseline compared with non‐obese, and are at risk of further reduction during anesthesia which may lead to atelectasis development, decreased lung compliance, and oxygenation difficulties.^25^, ^26^, ^27^ Although an intraoperative open lung approach, which may include lung recruitment maneuvers and PEEP titration, improves respiratory mechanics during anesthesia, positive airway pressures are lost following extubation and the beneficial effects from intraoperative ventilation approach on respiratory mechanics are attenuated.^28^ Ferrando et al. hypothesized that the loss of positive airway pressure following extubation may lead to the rapid development of atelectasis formation and that HFNO would not provide enough positive airway pressures to inflate them if they had already developed, but may prevent them if applied immediately upon extubation in the operation room.^13^ Although they found that immediate HFNO after extubation improved postoperative oxygenation, transportation with HFNO is currently not feasible in clinical practice. Furthermore, they administered a high dose of Fentanyl at anesthesia induction (5 µg kg^−1^) and maintained anesthesia with a Fentanyl infusion (3 µg kg^−1^ h^−1^) for approximately 2 h. Considering the context‐sensitive half‐life of Fentanyl,^29^ their results may thus only apply to obese patients with considerable remaining opioid effects in the PACU. In our study, a 2 µg kg^−1^ single dose of Fentanyl was administered at induction, and anesthesia was maintained with Remifentanil which has a short context‐sensitive half‐life^30^ that facilitates rapid recovery and may be more in line with current practice.

Corroborating the results of our study, a recently published investigation reported that HFNO failed to improve postoperative oxygenation compared with conventional oxygen therapy up to 6 h following bariatric surgery.^31^ However, oxygenation assessed by PaO_2_/FiO_2_ ratio was a secondary outcome variable in this trial, and FiO_2_ was calculated, not measured, for controls who received conventional low‐flow oxygen therapy. The delivered FiO_2_ with low‐flow devices is highly variable depending on the degree of room air entrainment^15^ and estimations will be inaccurate, hampering the interpretation of these results. Furthermore, they used a weaning protocol for oxygen therapy which yielded rather different levels of oxygen supplementation between groups over time which may have caused bias. For these reasons, FiO_2_ was not calculated in our study. Instead, similar levels of oxygen supplementation were achieved by targeting a pre‐specified initial peripheral hemoglobin saturation goal. Oxygen supplementation was then fixed allowing for PaO_2_ to be analyzed in isolation.

There was no difference in PaCO_2_ between groups. HFNO does not provide pressure support, and this finding was expected.^31^ However, previously diagnosed OSAS was uncommon in this study, and no patient had a prior diagnosis of obesity‐related hypoventilation which limits generalization to these populations. It is possible that patients with OSAS would benefit more from postoperative HFNO compared with patients without OSAS, although continuous positive airway pressure support should be regarded as the first‐line method for postoperative respiratory support in these patients.^32^, ^33^ If patients with OSAS do not tolerate CPAP, or this is not available postoperatively, HFNO may be an appropriate alternative although further research is warranted.

The findings of this study should be interpreted with its limitations. First, the single‐center study design and the homogenous population decrease external validity. Second, although the primary outcome was unlikely to be biased, this cannot be entirely excluded due to the unblinded design. Third, the small sample size decreases precision and the use of a convenience sample may increase the risk of type II error. However, the post hoc analysis determined that the sample size was adequate to detect a clinically relevant 20% difference in the primary outcome. Furthermore, the 95% CI provides additional information regarding the adequacy of statistical power. Fourth, closed mouth breathing leads to higher airway pressure during HFNO‐therapy.^22^ We did not instruct patients how to breathe, and the difference between groups may have increased if the patients had been instructed to breathe through their noses. However, due to patient compliance issues related to lingering effects of anesthetic agents, it would be very challenging, if not impossible to achieve strict nose breathing for longer periods of time in early postoperative care. Hence, we chose a pragmatic approach likely to simulate the effect of postoperative HFNO in clinical practice. Fifth, changes in lung volumes, spirometry, or other measures of respiratory mechanics that may not have been detected by parameters of oxygenation alone were not registered. Finally, we did not study long‐term respiratory or patient‐centered outcomes. However, our results may inform the design of such studies which are currently underrepresented in scientific literature.

## 5 CONCLUSIONS

In obese patients, HFNO did not improve short‐term postoperative oxygenation compared with standard low‐flow oxygen treatment after general anesthesia for laparoscopic bariatric surgery. These results do not support the generalized use of postoperative HFNO in obese patients. Further research is warranted to determine long‐term patient‐centered outcomes, especially in obese patients with a high risk of postoperative pulmonary complications.

## AUTHOR CONTRIBUTIONS

**Jacob Rosen**: Formal analysis; investigation; methodology; visualization; writing—original draft; writing—review & editing. **Peter Frykholm**: Conceptualization; investigation; methodology; supervision; writing—review & editing. **Diddi Fors**: Conceptualization; investigation; methodology; supervision; writing—review & editing.

## CONFLICTS OF INTEREST

Jacob Rosén and Peter Frykholm declare that they have no conflict of interest. Diddi Fors has received travel funding to participate in a scientific symposium on HFNO sponsored by Armstrong Medical Ltd, although not related to this study.

## DISCLOSURES

The corresponding author affirms that this manuscript is an honest, accurate, and transparent account of the study being reported; that no important aspects of the study have been omitted; and that any discrepancies from the study as planned (and, if relevant, registered) have been explained

## TRANSPARENCY STATEMENT

We confirm that this manuscript is an honest, accurate, and transparent account of the study being reported; that no important aspects of the study have been omitted; and that any discrepancies from the study as planned (and, if relevant, registered) have been explained.

## Data Availability

The data that support the findings of this study are available on request from the corresponding author. The data are not publicly available due to privacy or ethical restrictions.

## References

[1] Lindberg P , Gunnarsson L , Tokics L , et al. Atelectasis and lung function in the postoperative period. Acta Anaesthesiol Scand. 1992;36:546‐553. 1514340 10.1111/j.1399-6576.1992.tb03516.x

[2] Magnusson L , Zemgulis V , Wicky S , Tydén H , Thelin S , Hedenstierna G . Atelectasis is a major cause of hypoxemia and shunt after cardiopulmonary bypass: an experimental study. Anesthesiology. 1997;87:1153‐1163. 9366468 10.1097/00000542-199711000-00020

[3] Eichenberger A , Proietti S , Wicky S , et al. Morbid obesity and postoperative pulmonary atelectasis: an underestimated problem. Anesth Analg. 2002;95:1788‐1792. 12456460 10.1097/00000539-200212000-00060

[4] Ahmad S , Nagle A , McCarthy RJ , Fitzgerald PC , Sullivan JT , Prystowsky J . Postoperative hypoxemia in morbidly obese patients with and without obstructive sleep apnea undergoing laparoscopic bariatric surgery. Anesth Analg. 2008;107:138‐143. 18635479 10.1213/ane.0b013e318174df8b

[5] Fernandez‐Bustamante A , Frendl G , Sprung J , et al. Postoperative pulmonary complications, early mortality, and hospital stay following noncardiothoracic surgery: a multicenter study by the perioperative research network investigators. JAMA Surg. 2017;152:157‐166. 27829093 10.1001/jamasurg.2016.4065 PMC5334462

[6] Serpa Neto A , Hemmes SN , Barbas CS , et al. Incidence of mortality and morbidity related to postoperative lung injury in patients who have undergone abdominal or thoracic surgery: a systematic review and meta‐analysis. Lancet Respir Med. 2014;2:1007‐1015. 25466352 10.1016/S2213-2600(14)70228-0

[7] Canet J , Gallart L . Postoperative respiratory failure: pathogenesis, prediction, and prevention. Curr Opin Crit Care. 2014;20:56‐62. 24240985 10.1097/MCC.0000000000000045

[8] Squadrone V , Coha M , Cerutti E , et al. Continuous positive airway pressure for treatment of postoperative hypoxemia: a randomized controlled trial. JAMA. 2005;293:589‐595. 15687314 10.1001/jama.293.5.589

[9] Futier E , Paugam‐Burtz C , Godet T , et al. Effect of early postextubation high‐flow nasal cannula vs conventional oxygen therapy on hypoxaemia in patients after major abdominal surgery: a French multicentre randomised controlled trial (OPERA). Intensive Care Med. 2016;42:1888‐1898. 27771739 10.1007/s00134-016-4594-y

[10] Corley A , Bull T , Spooner AJ , Barnett AG , Fraser JF . Direct extubation onto high‐flow nasal cannulae post‐cardiac surgery versus standard treatment in patients with a BMI ≥30: a randomised controlled trial. Intensive Care Med. 2015;41:887‐894. 25851385 10.1007/s00134-015-3765-6

[11] Zoremba M , Kalmus G , Begemann D , et al. Short term non‐invasive ventilation post‐surgery improves arterial blood‐gases in obese subjects compared to supplemental oxygen delivery ‐ a randomized controlled trial. BMC Anesthesiol. 2011;11:10. 21605450 10.1186/1471-2253-11-10 PMC3117807

[12] Neligan PJ , Malhotra G , Fraser M , et al. Continuous positive airway pressure via the Boussignac system immediately after extubation improves lung function in morbidly obese patients with obstructive sleep apnea undergoing laparoscopic bariatric surgery. Anesthesiology. 2009;110:878‐884. 19293693 10.1097/ALN.0b013e31819b5d8c

[13] Ferrando C , Puig J , Serralta F , et al. High‐flow nasal cannula oxygenation reduces postoperative hypoxemia in morbidly obese patients: a randomized controlled trial. Minerva Anestesiol. 2019;85:1062‐1070. 30994312 10.23736/S0375-9393.19.13364-0

[14] Rosén J , Frykholm P , Fors D . High‐flow nasal cannula versus face mask for preoxygenation in obese patients: a randomised controlled trial. Acta Anaesthesiol Scand; n/a. 2021 Nov;65:1381‐1389. 10.1111/aas.13960 34309839

[15] Wettstein RB , Shelledy DC , Peters JI . Delivered oxygen concentrations using low‐flow and high‐flow nasal cannulas. Respir Care. 2005;50:604‐609. 15871753

[16] Pasquier EK , Andersson E . Pulmonary recruitment maneuver reduces pain after laparoscopic bariatric surgery: a randomized controlled clinical trial. Surg Obes Relat Dis. 2018;14:386‐392. 29290563 10.1016/j.soard.2017.11.017

[17] Talab HF , Zabani IA , Abdelrahman HS , et al. Intraoperative ventilatory strategies for prevention of pulmonary atelectasis in obese patients undergoing laparoscopic bariatric surgery. Anesth Analg. 2009;109:1511‐1516. 19843790 10.1213/ANE.0b013e3181ba7945

[18] Futier E , Constantin J‐M , Pelosi P , et al. Noninvasive ventilation and alveolar recruitment maneuver improve respiratory function during and after intubation of morbidly obese patients: a randomized controlled study. Anesthesiology. 2011;114:1354‐1363. 21478734 10.1097/ALN.0b013e31821811ba

[19] Rosén J , Frykholm P , Fors D . High‐flow nasal cannula versus face mask for preoxygenation in obese patients: A randomised controlled trial. Acta Anaesthesiol Scand. 2021;65:1381‐1389. 10.1111/aas.13960 34309839

[20] Groves N , Tobin A . High flow nasal oxygen generates positive airway pressure in adult volunteers. Aust Crit Care. 2007;20:126‐131. 17931878 10.1016/j.aucc.2007.08.001

[21] Ritchie JE , Williams AB , Gerard C , Hockey H . Evaluation of a humidified nasal high‐flow oxygen system, using oxygraphy, capnography and measurement of upper airway pressures. Anaesth Intensive Care. 2011;39:1103‐1110. 22165366 10.1177/0310057X1103900620

[22] Parke R , McGuinness S , Eccleston M . Nasal high‐flow therapy delivers low level positive airway pressure. Br J Anaesth. 2009;103:886‐890. 19846404 10.1093/bja/aep280 PMC2777940

[23] Riera J , Pérez P , Cortés J , Roca O , Masclans JR , Rello J . Effect of high‐flow nasal cannula and body position on end‐expiratory lung volume: a cohort study using electrical impedance tomography. Respir Care. 2013;58:589‐596. 23050520 10.4187/respcare.02086

[24] Corley A , Caruana LR , Barnett AG , Tronstad O , Fraser JF . Oxygen delivery through high‐flow nasal cannulae increase end‐expiratory lung volume and reduce respiratory rate in post‐cardiac surgical patients. Br J Anaesth. 2011;107:998‐1004. 21908497 10.1093/bja/aer265

[25] Damia G , Mascheroni D , Croci M , Tarenzi L . Perioperative changes in functional residual capacity in morbidly obese patients. Br J Anaesth. 1988;60:574‐578. 3377932 10.1093/bja/60.5.574

[26] Pelosi P , Croci M , Ravagnan I , et al. The effects of body mass on lung volumes, respiratory mechanics, and gas exchange during general anesthesia. Anesth Analg. 1998;87:654‐660. 9728848 10.1097/00000539-199809000-00031

[27] Lundquist H , Hedenstierna G , Strandberg A , Tokics L , Brismar B . CT‐assessment of dependent lung densities in man during general anaesthesia. Acta Radiol Stockh Swed 1987: 1995;36 626‐632. 8519574

[28] Ferrando C , Soro M , Unzueta C , et al. Individualised perioperative open‐lung approach versus standard protective ventilation in abdominal surgery (iPROVE): a randomised controlled trial. Lancet Respir Med. 2018;6:193‐203. 29371130 10.1016/S2213-2600(18)30024-9

[29] Hughes MA , Glass PS , Jacobs JR . Context‐sensitive half‐time in multicompartment pharmacokinetic models for intravenous anesthetic drugs. Anesthesiology. 1992;76:334‐341. 1539843 10.1097/00000542-199203000-00003

[30] Kapila A , Glass PS , Jacobs JR , et al. Measured context‐sensitive half‐times of remifentanil and alfentanil. Anesthesiology. 1995;83:968‐975. 7486182 10.1097/00000542-199511000-00009

[31] Fulton R , Millar JE , Merza M , et al. Prophylactic postoperative high flow nasal oxygen versus conventional oxygen therapy in obese patients undergoing bariatric surgery (OXYBAR Study): a pilot randomised controlled trial. Obes Surg. 2021;31:4799‐4807. 34387826 10.1007/s11695-021-05644-y

[32] Subramani Y , Singh M , Wong J , Kushida CA , Malhotra A , Chung F . Understanding phenotypes of obstructive sleep apnea: applications in anesthesia, surgery, and perioperative medicine. Anesth Analg. 2017;124:179‐191. 27861433 10.1213/ANE.0000000000001546 PMC5429962

[33] Chung F , Nagappa M , Singh M , Mokhlesi B . CPAP in the perioperative setting: evidence of support. Chest. 2016;149:586‐597. 26469321 10.1378/chest.15-1777 PMC5831563

